# ‘Policing’ a pandemic: Garda wellbeing and COVID-19

**DOI:** 10.1017/ipm.2020.70

**Published:** 2020-05-28

**Authors:** L. Rooney, F. McNicholas

**Affiliations:** 1 Department of Child & Adolescent Psychiatry, University College Dublin, Dublin, Ireland; 2 UCD Institute of Criminology and Criminal Justice, University College Dublin, Dublin, Ireland; 3 Our Lady’s Children’s Hospital, Crumlin, Ireland; 4 Lucena Clinic Services, Rathgar, Dublin, Ireland; 5 School of Medicine and Medical Science, Geary Institute, University College Dublin, Dublin, Ireland

**Keywords:** COVID-19, Pandemic, Policing, The Garda Síochána, psychological support needs

## Abstract

In response to the global pandemic COVID-19, the Irish government has called upon the Garda Síochána to implement an unparalleled mode of policing to mitigate and contain the spread of the Coronavirus. Studies investigating smaller scale epidemics, such as Severe Acute Respiratory Syndrome (SARS), indicate that staff at the frontlines of an outbreak are exposed to an insuperable amount of stress and experience increased psychological morbidities as a result. Furthermore, research not only indicates that heighted levels of psychological distress are an occupational hazard associated with the law enforcement profession, but that members of the Garda Síochána feel their mental health needs are largely unmet by their organisation. Given the pandemic’s propensity to expose officers to indeterminate echelons of physical and psychological threat; there has never been a more appropriate time to explore the potential burdens associated with ‘policing’ a pandemic, question the governments capacity to address the psychological support needs of frontline professionals, and plan future research for best practice.

The duties of the Garda Síochána stretch far beyond ‘traditional’ modes of policing, such as solving crime and law enforcement. Indeed, a considerable amount of their time is spent implementing harm reduction measures which aim to protect vulnerable members of the community, specifically, individuals with mental health and addiction issues, homeless people, children, and the elderly (Conway, 2009; Mental Health Commission & An Garda Síochána, 2009). In the wake of the COVID-19 pandemic, the Irish government has called upon Garda members to impose a unique mode of harm reduction and ‘police’ for the spread of a fatal pandemic. With an entire population rendered vulnerable by COVID-19, this occasional piece seeks to explore the extraordinary mission the Gardai Síochána have been tasked, consider the potential burdens associated with it and make recommendations for future research and practice.

## COVID-19 the Irish context

The first case of COVID-19 in the Republic of Ireland was confirmed on February 29, 2020. Almost 2 weeks later, on March 11, the Department of Health reported the first coronavirus fatality. In the weeks that followed, the Irish government implemented a series of public safety measures which aimed to contain virus spread. Namely, on March 12, all schools, colleges and childcare facilities were closed. Three days later, on March 15, the government requested that all pubs, clubs and entertainment venues close their doors. Finally, after a period of steady increases in confirmed cases and fatalities, the country entered full lockdown on March 27 under the advisement of the National Public Health Emergency Team. Full lockdown required that people remain in their homes at all times, with the exception of the measures outlined in Table 1.

**Table 1.** Summary table of circumstances in which persons may leave their home during lockdown. Released by the government on March 27, 2020

Lockdown measures (www.gov.ie, 2020a)
1. Travel to and from work where the work is considered an essential service.
2. Working in an essential shop, bank or post office.
3. Buying food, medicines and other health products for yourself, your family or someone who is vulnerable or ‘cocooning’.
4. Attending medical appointments
5. Vital family reasons including caring for children, elderly or vulnerable people but excluding social family visits.
6. Exercise within 2 km of your house.

The Garda Síochána has been an integral cog in the government’s response to the COVID-19 crisis. The force has had to embrace a series of changes to their professional duties over a very short time frame. Initially, system adjustments made by the Garda Síochána aimed to ensure their availability to assist vulnerable groups in the community. This restructure involved new roster arrangements, postponing retirements, allocating frontline duties to 319 Gardaí recruits, commissioning 210 extra vehicles and establishing a specialist COVID-19 unit (Department of Justice and Equality, 2020; Sheehy, 2020). However, a steady worsening of the problem necessitated a significant increase in public surveillance to keep people safe. Specifically, in the lead up to the Easter bank holiday weekend, government officials raised concerns that citizens might ignore lockdown measures to flock to popular tourist hotspots, beaches and holiday homes to enjoy a spell of fine weather. Accordingly, the decision was made to increase Garda Powers and implement a graduated policing plan to shift social distancing measures from an informal (moral) practice to a formal (legal) one. On April 7, it was signed into law that any person in breach of government restrictions on movement would be committing an offence under Section 31A of the Health Act of 1947 and could face a maximum of 6 months in prison or a fine of €2500 (Gov.ie, 2020b). Gardaí were given the power to direct people to comply with the new legislation, request the name and address of persons suspected of being in breach of lockdown guidelines, and three different powers of arrest. Such legislative changes are quite novel in that Gardaí will be operating under public health legislation and not criminal justice legislation as they usually do. As a result, Garda visibility has ramped up considerably with 2500 Garda members manning checkpoints on major roads across the country and patrolling cities, parks and beauty spots to ensure public compliance. As per the Taoiseach, Leo Varadkar’s, announcement on April 10, these measures will remain in place until the lockdown has been lifted, which remains unknown at this time (rte.ie, 2020a).

## Resistance, threat and burden

At the beginning of 2020, it was unthinkable that the Gardaí could send a person home just for walking outside a 2-km radius from their house. Yet, despite the plethora of rapid changes to the nation’s social freedoms, the majority of the public have complied with lockdown measures. This is evidenced by aerial photographs taken by the Garda Air Support Unit of empty streets, parks and tourist spots (Meneely, 2020), and energy consumption data to show that people are in fact staying at home (rte, 2020b). However, media reports suggest that Gardaí have been met with varying levels of resistance and threat when enforcing government restrictions on movement. Such resistance has ranged from people ignoring lockdown regulations and embarking on non-essential travel – to officers being spat and coughed at by members of the public (Reynolds, 2020). To keep officers safe from spit and cough assaults, frontline personnel have been issued with specialist Personal Protective Equipment (PPE) known as ‘spit hoods’ along with other items, such as goggles, masks, gloves and antiseptic wipes (ibid). These heinous attacks on Gardaí are extremely dangerous in terms of infection control and are likely very upsetting for those who have been targeted. Thus, it is imperative that Garda officials are mindful of other threats to officer wellbeing that can be equally arduous, but more covert in their manifestation, that of psychological distress. Just as it has done with the general public, COVID-19 has exposed the Gardaí to a myriad of environmental stressors that have the potential to jeopardise their psychological wellbeing. However, the difference is that Garda members not only put their physical health and mortality at risk when they go to work every day, but must do so whilst also having to cope with a new class of anxieties and responsibilities associated with ‘policing’ a pandemic.

Research investigating smaller scale epidemics, such as severe acute respiratory syndrome (SARS), and emerging scholarship concerning COVID-19, demonstrates that the unequalled amount of stress experienced by essential staff working at the frontlines of the outbreak is associated with increased psychological morbidities (Huang *et al.* 2004; Tsamakis *et al.* 2020). For instance, in the early stages of the SARS outbreak, when virus spread was at its most rapid, healthcare professionals reported feelings of extreme vulnerability and uncertainty, a significant threat to life, and developed symptoms of anxiety (both somatic and cognitive) (Huang *et al.* 2004; Tsamakis *et al.* 2020). Current research exploring the experience of healthcare professionals dealing with COVID-19 in China and Greece details similar findings. Frontline personnel report feeling overwhelmed by the potential to be scrutinised by the press, frustrated by uncooperative patients who resist adherence to safety instructions, and fear of contracting the virus themselves only to expose their loved ones (Tsamakis *et al.* 2020). Prolonged use of PPE makes it difficult for workers to breathe, access water and use the toilet, causing additional physical and mental fatigue (Chen *et al.* 2020; Tsamakis *et al.* 2020). Furthermore, risks to the psychological wellbeing of frontline workers are further exacerbated by reports of PPE shortages and defective equipment (Sim, 2020). It is also worth noting that most of the scholarship in the area focuses on the experiences of healthcare professionals, whilst the perspective of the police and other criminal justice professionals (i.e. prison officers, probation officers), heavily involved in the emergency response, has been largely overlooked. The absence of such commentary suggests a collective prioritisation by decision-makers, academics and the public to explore and improve supports for frontline staff with health-focused roles whilst failing to consider the needs of others such as law enforcement. Moreover, the lack of a concerted effort to explore, understand and implement supports for criminal justice professionals during COVID-19 means that many essential staff may be experiencing psychological difficulties that could worsen considerably if they remain undetected and untreated.

The past couple of months has seen an upsurge in social media and newspaper coverage expressing the nation’s justified gratitude to healthcare staff for their incredible courage and sacrifice amidst the COVID-19 crisis. Although a great deal of attention has also been given to increasing Garda Powers and the public response to these directives, there has been very little acknowledgement of the Garda Síochána’s bravery or of the risk their job entails. The absence of such media homage suggests that the Gardaí represent a group of frontline professionals whose heroism not only remains unsung, but whose contribution is perhaps taken for granted. Media tributes are relevant to this discussion as research indicates that expressions of social support and gratitude help individuals to develop psychological resources that mitigate environmental stressors (Eaton *et al.* 2014; Glasgow *et al.* 2016). Moreover, the link between gratitude and psychological wellbeing has also been identified amongst groups of first responders following terrorist attacks and natural disasters. For instance, research investigating police officer’s ability to successfully adapt to stressors and maintain psychological wellbeing following Hurricane Katrina revealed that gratitude helped to alleviate symptoms of post-traumatic stress disorder (PTSD) (McCanlies *et al.* 2014). Similarly, strong feelings of gratitude were associated with lower levels of psychological distress amongst first responders following the terrorist attacks on September 11, 2011 (Fredrickson *et al.* 2003). Given the minimal amount of media gratitude expressed to the Garda Síochána for their role in COVID-19, this research not only infers that officers are excluded access to certain sociocultural factors that mitigate environmental stressors but highlights an additional level of vulnerability that should be considered when developing support services for the Gardaí.

Even in the absence of a global pandemic, police work is regarded as one of the most stressful occupations. As a result, it is associated with serious challenges to mental and emotional wellbeing (Violanti & Paton, 1999; Liberman *et al.*, 2002). Officers are not only exposed to the full gamut of social traumas (i.e. extreme poverty, addiction, violence, death and tragedy), but are forced to contemplate their own mortality when faced with potentially life-threatening situations (Miller, 1995; Bartol & Bartol, 2008). Moreover, the death of a colleague in the line of duty is cited as by far the most ‘psychologically destabilizing’ experience for members of the force (Henry, 2004; Miller, 2007: 14). Research indicates that exposure to such occupational stressors can negatively impact the professional and personal lives of officers. For instance, when compared to other public servants, the police report elevated rates of alcohol misuse, divorce, anxiety and depression, suicide, and burnout (Violanti & Paton, 1999; Tanigoshi, *et al.* 2008).

In recent years, the Garda Síochána has enhanced its psychological support services. In 1994, the Peer Support Program was established, which activates the deployment of a trained Peer Supporter to assist officers following a traumatic event. This was followed by the introduction of a confidential 24-hour counselling line in 2016 (Oirachtas.ie 2020). However, despite the implementation of these services, research indicates that Garda members feel that these programmes are not only ill-equipped to meet their support needs, but that the delivery of these resources are both inconsistent and unreliable (Fallon, 2018). These sentiments are most acutely felt when it comes to debriefing officers following a traumatic event. For instance, one officer stated, ‘I spent 9 years in a busy Dublin station and not once was I contacted by a welfare officer following traumatic incidents’ (ibid: 98). Another commented ‘Far too much box ticking for services without real concern for the affected members welfare’ (ibid: 111). Fallon concludes that ‘An Garda Síochána is a cauldron for adversity in relation to trauma and wellbeing’, with one in six officers potentially meeting criteria for PTSD, one in four experiencing significant levels of trauma-induced distress and impairment, and a high rate of completed suicide amongst its members (ibid:9). Indeed, the implementation of support service’s by the Garda Síochána is a step in the right direction in terms of promoting occupational mental health. However, the usefulness of these programmes must be called into question when personnel perceive them as tokenistic and are sceptical about their very existence. Given the overwhelming body of evidence emphasizing the relationship between police work and impaired psychological health, coupled with domestic research to show that many of the Gardaí are ‘walking wounded’; it is imperative that Garda officials begin to prioritise the psychological wellbeing of their members and provide them with the resources they deserve.

The coronavirus has forced the Gardaí Síochána to take on an extraordinary mode of policing in the name of public safety and infection control. Like many other essential workers, their position at the forefront of the pandemic could expose them to increased levels of death and trauma, not just amongst the general population but within their organisation. It is our position that there has never been a more fitting time to question the government’s preparedness and capacity for meeting the psychological support needs of frontline criminal justice professionals whom we continue to rely on so heavily in this time of crisis.

## Current position and future directions

At baseline, the Gardaí are exposed to an increased level of psychological distress. This is compounded by a perceived lack of support on behalf of the organisation when it comes to addressing matters of mental health and wellbeing (Fallon, 2018). Although it is impossible to know the just how COVID-19 will impact the physical and psychological health of the Garda Community, what is certain, is that officer wellness has never be more important than it is at this moment and in the weeks that follow.

It is extremely unfortunate that very few, if any, interventions addressing the psychological support needs of frontline professionals in the midst of a global pandemic have been evaluated and deemed effective for use. However, a lack of evidence-based practice does not mean that nothing can be done to help and support essential staff. To best determine a way forward, policymakers and senior management can consult literature surrounding the provision of mental health support during natural disasters and national emergencies (see American Red Cross, 2005; World Health Organisation, 2020), along with broader theory and research regarding first responder occupational mental health and wellbeing. There is no shortage of scientific evidence identifying the significant role that psychological interventions play in helping the police cope with stress and trauma (see, Chopko & Schwartz, 2009; Andersen *et al.*, 2015; Papazoglou & Tuttle, 2018). For instance, inquiries into the provision of urgent psychological support in the wake of a traumatic event attests to the benefits of Critical Incident Stress Management (CISM) systems (Flannery, 1999; Everly *et al.* 2000; Guenthner, 2012). CISM is defined as a ‘formal, highly structured and professionally recognised process for helping those involved in a critical incident to share their experiences, vent emotions, learn about stress reactions and symptoms and given referral for further help if required’ (CISM Network Ireland, 2020). Findings suggest that participants benefit from CISM systems by developing a range of strategies that work to mitigate psychological distress and promote mental wellbeing (Everly *et al.* 2002). Recent research investigating proactive training programmes specifically designed to help first responders cope with future critical incidents have also yielded promising results. For example, Mindfulness-Based Resilience Training has been shown to reduce perceived stress, depression, anxiety, disrupted sleep, burnout, self-reported anger and PTSD symptoms in police officers (Christopher et al 2016, 2018; Grupe *et al.*, 2019). Moving forward, the Department of Justice should strongly consider piloting and scaling up a CISM system or similar programme, not just for Garda members, but for all its personnel (i.e. prison officers, probation officers, legal counsel, etc). In the interim, to combat and protect the Gardaí from psychological distress during the COVID-19 pandemic, it is recommended that the force is reminded of the supports that are currently available to them (i.e. counselling support line, Peer Support Program). More importantly, it is advised that a concerted effort is made by Garda officials to ensure these supports are diligently implemented, fully operational and easily accessible so that they may be of benefit to those who may need them.

Finally, in the months that follow, it is important that government officials, decision-makers and scholars use COVID-19 as an opportunity to collate experiential knowledge and insights from those working on the frontline (Gardaí, healthcare professionals, shopkeepers, bank tellers, etc.). Future research exploring the opportunities and challenges associated with maintaining mental health equilibrium and psychological wellbeing will help to identify occupational support needs, inform crucial policy and practice, and contribute to Ireland’s overall level of preparedness for future national emergencies.

## Financial support

This article received no specific grant from any funding agency, commercial or not-for-profit sectors.

## Conflict of interest

The authors have no conflict of interest to disclose.

## Ethical standards statement

The authors assert that all procedures contributing to this work comply with the ethical standards of the relevant national and institutional committee on human experimentation with the Helsinki Declaration of 1975, as revised in 2008. The authors assert that ethical approval was not required for publication of this manuscript.
